# Chile’s 2021 constitutional neurorights amendment: a cognitive sovereignty preliminary conceptual framework for non-traditional security analysis

**DOI:** 10.3389/fpubh.2026.1843113

**Published:** 2026-09-03

**Authors:** Karolina Zhukoff

**Affiliations:** Georgia Institute of Technology, Sam Nunn School of International Affairs, Atlanta, Georgia

**Keywords:** Chile, cognitive sovereignty, global south, neurorights, neurotechnology governance, non-traditional security, post-colonial statehood, technological asymmetry

## Abstract

Chile’s 2021 constitutional amendment on neurorights, the first such provision anywhere in the world, is widely characterized in the literature as a rushed, conceptually underdeveloped instance of liberal rights extension. This article proposes an alternative framing. Examined through the intersecting lenses of non-traditional security (NTS) theory, international law, and the political history of post-colonial statehood and technological dependency, this article challenges this view and proposes that the 2021 constitutional amendment can be functionally interpreted as acquiring the structure of *cognitive sovereignty*, defined here as the state’s independent authority over the mental and identity-related domains of its population against disproportionate foreign neurotechnological influence. Three analytical dimensions support this functional reading. First, the amendment’s placement within Chile’s restrictive bioethical architecture, − which severely constrains bodily autonomy while constitutionally elevating neural governance, − is structurally inconsistent with a liberalizing rights logic and is more coherently read as an extension of conservative state authority over the cognitive domain. Second, Chile’s history of foreign intervention and the Pinochet regime’s systematic use of psychological methods to fracture political consciousness, constitutes a historical context within which the amendment acquires cognitive sovereignty significance, even if history did not directly motivate its drafters. Third, Chile’s structural position as a consumer rather than producer of neurotechnology, operating under the conditions of technological asymmetry, provides geopolitical context within which the amendment may function as a reverse regulatory signal toward technologically dominant actors, regardless of whether it was designed as such. Taken together, these three contextual dimensions challenge the dismissal of the amendment as conceptually underdeveloped and suggest that, when functionally interpreted through cognitive sovereignty lens, neurorights may acquire strategic significance as NTS instruments – a reading that complements rather than replaces the human-rights framing. The article concludes by exploring the conditions under which constitutional neurorights may be adopted by other technologically peripheral states as for cognitive sovereignty purposes, with implications for global neurogovernance.

## Introduction

When mid-twentieth-century experiments demonstrated that electrical stimulation could modulate the behavior and affective states of animals, they marked an early empirical threshold: it had become technically plausible to intervene directly in the mechanisms of the mind. The thinking subject is necessarily a political subject, ‘the very presence of mind is a political presence,’ and the possibility of altering neural function was interpreted not merely as a biomedical advance but as a challenge to the foundations of liberal political order (1). Cold War anxieties intensified around reports that the Soviet government was developing forms of ‘brain warfare,’ prompting liberal democracies to position universal human rights, particularly freedom of thought, as a protective umbrella against cognitive interference (1, 2). Yet the more consequential question today is whether the modern expanding reach of neurotechnology has reproduced that threat structure at a global scale.

The starting proposition is that the cognitive domain, previously protected through instruments designed to address interference operating at the level of the informational environment infrastructure and psychological operations, is now subject to interference capable of reaching the cognitive substrate directly, which renders the protection those instruments afforded structurally insufficient. Neuroscience advancements suggest it may now be necessary to address *state cognitive security*, whereby the cognitive layer of the state’s population could be viewed as domain of state authority, the mind as its territory, and that territory as one that neurotechnology is now capable of reaching. As such, cognitive security arises as a pressing concern at the individual level, and - in the context of the broader definition of neurotechnology as a brain model capable of predictive and modulative intervention - extends to the collective and thus state levels, giving rise to *state cognitive sovereignty* claim. Neurotechnology reaches directly into the substrates of decision-making, intention, and identity, at both individual and collective scales, the cognitive foundation through which political participation is exercised.

At the foundation of this work lies a problem that international studies have not yet adequately addressed. The security implications of neurotechnological global asymmetry remain underarticulated in the literature, and neurorights, widely discussed in ethical and legal scholarship regarding whether they enhance individual protection or merely replicate existing human-rights guarantees, have not been examined as instruments of state sovereign authority in response to NTS challenges. This article proposes that when Chile’s 2021 constitutional amendment is viewed not through a human-rights lens but through the lens of global technological power asymmetries, it acquires additional analytical security significance. This article does not claim that Chilean policymakers consciously framed neurorights as sovereignty instruments; rather, it argues that the amendment exhibits the structural characteristics of such a sovereignty claim when situated within its historical and geopolitical context. NTS theory is employed here as the framework through which individual-level cognitive risks become legible as state-level and international security concerns, tracing the path from personal neural vulnerability to collective political identity and, ultimately, to sovereign authority, as demonstrated through the case of Chile. Under this reframing, neurorights may be interpreted as a sovereignty-oriented response to NTS challenges introduced by neurotechnology, rather than a liberal rights expansion. The article does not claim to fully demonstrate this thesis; rather, it prepares the analytical ground for future empirical and theoretical development. This analysis proceeds from the assumption that the relevant threats are structurally rooted in neurotechnological asymmetry, with dominant capabilities concentrated in a small number of states and exercised through both private and state actors operating within those states’ jurisdictions.

The article does not contest the 2021 constitutional amendment genealogy, which originated from collaboration between Senator Guido Girardi and an international network led by Rafael Yuste. In Yuste’s formulation (3, 4) neurorights are framed explicitly in terms of universal human dignity, individual cognitive liberty, mental privacy, and informed consent in the face of emerging neurotechnology – not in terms of sovereignty, post-colonial legacy, or technological asymmetry. Rather, it advances a different claim; the amendment, whatever is stated rationale, exhibits the structural characteristics of a cognitive sovereignty assertion when situated within Chile’s historical and geopolitical context. A legal provision may acquire political functions that its original promoters did not explicitly intend. The analytical move this article makes is therefore ex post and functional, as it demonstrates what the amendment *does*, rather than what it was designed to do. The distinction between origin and possible later function does not render the two mutually exclusive.

The article generates several policy implications that follow from this theoretical framing. First, it contends that regulatory instruments need to be designed to make the cognitive layer explicitly a domain of protection from interference and uses the example of Chile. In connection with this, states which have the peripheral positioning, such as Chile, within the technological power asymmetries are expected to respond to this necessity further potentially through a similar *right-based* pathway. Neurotechnology governance cannot be negotiated as a purely technical or ethical matter, but must also be considered a geopolitical one, and frameworks that ignore this will reproduce the fragmentation already visible in other dimensions of digital governance. Because of the difficult-to-identify dual-use threshold for neurotechnology, policy must rely on effect-based regulation that addresses what a technology does to cognition, rather than defining based on the intended use. Finally, the claim of this article should lay the foundation for the further theorizing of cognitive sovereignty which could emerge as a norm with a “double edge.” If protections are recognized, it could provide a legitimate basis for states to protect their populations’ cognitive environments from foreign interference, but it could simultaneously justify domestic cognitive control. The international community would need to develop the equivalent of the sovereignty-vs.-human-rights distinction that already exists in other domains: distinguishing between a state’s legitimate protective claim over the cognitive conditions of collective political agency and its use of that claim to suppress internal dissent.

### Neurotechnology definition and cognitive scope

For the purpose of this article, neurotechnology is defined broadly. The narrow definition concerns direct neural interfaces - where the input is brain activity itself, recorded or stimulated at the level of the neural substrate, and the output is a measured or modulated neural state. The broader definition, which this article adopts, encompasses technologies whose input is a computational model of the brain - constructed from behavioral data, digital traces, and algorithmically inferred cognitive patterns - and whose output is predictive of mental states, attentional dispositions, and decision- making tendencies, enabling targeted influence on cognition before those mental states are consciously formed or behaviorally expressed. This definition encompasses brain-computer interfaces (BCIs), neuro-recording and stimulation devices, neuroimaging, as well as digital platforms and algorithmic systems whose primary mechanism of effect operates at the cognitive level (5).

Consistent with the broader approach adopted here, neurotechnology operates across both conscious and subconscious levels of cognition - or, in neuroscientific terms, across explicit and implicit processing (6). On this account, a decision-making process is considered conscious to the degree that each prompt within it is evaluated with a measurable degree of confidence - that is, to the degree that the subject is aware of the information being processed and the weight assigned to it (7). Neurotechnology-facilitated interventions that reach below the threshold of conscious awareness are structurally beyond the reach of informed consent. If it is beyond that reach, then at the individual level one becomes cognitively dispossessed, as something occurs outside the reach of one’s conscious comprehension.

The initial intake of environmental information is primarily mediated through conscious attention, though a concurrent stream of processing occurs below the threshold of awareness, meaning that the cognitive environment is being shaped through channels the individual neither monitors nor consciously controls (8). Subsequent stages of processing operate largely below conscious awareness, for example, in the encoding of memories and their retrieval back into conscious perception, through which prior experience continuously shapes the interpretation of present information. From a neuroscientific standpoint, memory is not merely archival - it is constitutive of present perception and anticipation of the future, as the brain continuously draws on encoded past experience to construct its representation of current reality and project forward from it (9, 10). The cognitive element of political identity, built as an autobiographical memory record, is not static but a dynamic one, in which the past is perpetually being built into the present and the future. The political significance of this trajectory becomes apparent at the point where neural processes involved in such processing are becoming technically accessible and predictable not only at the individual level but at the collective. Political psychology has examined the relationship between collective memory and political participation at both national and sub-national levels, and the degree to which shared experience shapes political identity and behavior (11). Chen et al. (12) provide evidence that shared experiences generate shared structure in neural activity across individuals. Shared experiences are reconstituted in an *imaginatory way* - which in neuroscience terms is a brain integrative network processing of input that builds an individual narrative. Shared narratives are co-created through social interaction in the pursuit of meaning - shared interpretation. When such interpretation is similar among individuals, it corresponds to neural responses traceable across the collective (13). When different people encode and later recall the same experience or in other words, narrative, their higher-order cortical representations - in the default mode – implicit network, − converge on common spatial patterns (12). Individual memory - autobiographic - is substantially responsible for preserving the stable narrative of oneself. It includes one’s shared collective environment, and thus one’s participation in it as, among other ways, a part of political community, and therefore may be understood to be organized at the level of collective neural structure. The collective cognitive world that political communities share suggests the existence of a neuronal substrate for shared political cognition – one that emerging neurological capabilities may render increasingly legible. If shared memories produce shared neural representation, and if that representation is the mechanism through which political identity, legitimacy, and allegiance are instantiated across a population, then the technical accessibility of individual cognition raises the theoretical possibility of collective cognition prediction, with implications - not yet empirically established - for the cognitive foundations through which the state is constituted as a representative, stable actor of a collective and through which individuals become political participants within it, whether at the level of conscious expression or subconscious formation.

For the purposes of this article, the systems producing the cognitive effects described above are predominantly designed, owned and operated by actors domiciled in technologically dominant state, while their effects operate within the populations of other states. The cross-border cognitive reach gives the sovereignty question analytical force which the 2021 constitutional amendment on its functional reading addresses.

## Theoretical framework: sovereignty, NTS, and the cognitive domain

### The analytical problem with sovereignty

Sovereignty is among the most contested concepts in international relations theory, and its deployment as an analytical tool requires definitional clarity and constraint. Constructivist accounts treat the shifting nature of sovereignty as unproblematic, arguing that as collective understandings of personhood, risk, and legitimate authority evolve, so too does what sovereignty means and who is entitled to invoke it. Under this view, the emergence of neurorights as a constitutional expression of cognitive self-governance is simply another instance of sovereignty’s ongoing renegotiation in response to new technological realities within international relations. This approach is criticized for risking the treatment of sovereignty as infinitely *fluid,* too malleable to function as a rigorous analytical instrument.

Stephen Krasner (14) offers an account of sovereignty as not a single coherent notion but a cluster of distinct dimensions that must be disaggregated for analytical clarity. These include international legal sovereignty (mutual recognition between states), Westphalian sovereignty (the exclusion of external actors from domestic authority structures), domestic sovereignty (the organization and effectiveness of internal authority), and interdependence sovereignty (the state’s capacity to regulate cross-border flows) (14). Each dimension can be present or absent independently of the others, which means a state may enjoy full formal recognition while its domestic authority is undermined by external actors, a condition highly relevant to the Chilean case. Katja Weber (15) insists that sovereignty must be treated as a legal absolute, as an invariant categorical constant, rather than as a quantitative attribute that states can possess in degrees. It must not be confused with ‘formal power,’ ‘legal freedom,’ ‘political independence,’ or ‘freedom of action,’ all of which admit of gradation (15). This definitional precision matters: a state may hold full international legal sovereignty while its practical capacity to govern its own domain is materially constrained by external actors. As will be shown, this gap between formal and effective sovereignty is the condition Chile occupies in the neurotechnology domain.

### Cognitive foundation as security domain

The individual cognitive foundation has become, first, legible as brain processes may now be describable with scientific precision and can be influenced through technological systems operating at scale. Second, it has become malleable in a qualitatively new way - reaching not only the products of conscious cognition through individual political expression, but the subconscious, as the neural processes through which political reality is perceived, identities within the state are formed, and allegiance to state authority is constructed - which may increasingly constitute a targetable vulnerability. The form of the novel cognitive security threat neurotechnology poses operates at deeper levels of neural processing, with the potential to affect the conditions under which perception itself operates. Rikli and Knappe (16) define cognitive security on the international scale as “a state and process in which undesired malign influence or manipulation is incapable of altering human cognition”. The recognition of deeper security concerns at the level of more advanced neurotechnological involvement has pushed the policymaking further, with the NATO’s Cognitive Security Act published in 2025 (53), placing cognitive security on the alliance’s formal agenda, and the US National Defense Authorization Act for Fiscal Year 2026 (59) mandating to create a task force to define cognitive warfare (54).

### Neurotechnological interference as a non-traditional security challenge

Cognitive security gives rise to cognitive sovereignty, defined as the state’s claim that the cognitive foundation through which collective political agency is constituted within the state – defined here as collective cognitive allegiance - falls within a domain of sovereign authority, subject to, inter alia, the norm of non-interference. This formulation draws on national collective identity theory to connect the individual to the collective and, through it, to the international (17). The national identity framework describes the mechanism of collective identification through which security interests are constituted (18) and through which, this article proposes, the state’s claim to sovereign authority over its population is grounded. The border that this sovereignty claim asserts is a cognitive one rather than territorial, but sovereign in the fundamental sense, as it delimits a domain within which external interference is not permitted and the penetration of which by foreign actors or foreign-controlled platforms constitutes a violation of sovereign coherence in a new territorial sense - the territory of its population’s mind. The cognitive foundation, the shared processes through which a political community is associated with, belonged to, maintained, reproduced, and imagined, is constituted through shared narratives, perceptions, and interpretive frameworks produced and upheld by the state. Nationalism is one illustration of this mechanism, though the argument of this article extends beyond it without claiming to analyze it fully. Rather than a merely political or historical phenomenon, nationalism, as O’Mara (19) demonstrates, is rooted in evolved neurocognitive and biological capacities for memory, imagination, group affiliation, and emotional bonding, capacities that are foundational to collective identification more broadly. Identification with the state through nationalism or national identity depends on the individual brain’s capacity to construct a coherent representation of the political and social world, a capacity grounded in prefrontal cortical systems that support episodic memory and imaginative projection (19). Through the collective exercise of these same capacities, shared across individuals inhabiting a common environment, that the fusion of individual and group identity is formed and sustained. Affective processes including pride, fear, and moral outrage that scale individual emotional responses to mass political communities are culturally mediated in group memory and narrative systems (19). Drawing on Benedict Anderson’s Imagined Communities (60), an imaginative projection of the national identity happens within its members community despite not actually meeting or knowing each other, via shared mental image of their communion and solidarity. Anderson captures nationalism as rooted in shared narratives and strengthened through mass communication. The ontological security theory (20) justifies the motivational logic for why states might treat the cognitive disruption as existential. States, like individuals, are driven to maintain a stable and continuous sense of identity, and when the cognitive conditions that reproduce that identity are threatened, the response is not merely defensive but constitutive of the state’s continued existence as a coherent political actor (20).

Non-traditional security (NTS) theory reconceptualizes security threats as those that originate outside the framework of interstate military conflict, arising instead from economic, environmental, societal, or technological sources that nonetheless destabilize the conditions under which states exercise authority and populations sustain collective life. A threat is non-traditional if it operates below or across the threshold of formal military confrontation, involves non-state actors as both threat sources and targets, and generates security pressures that states cannot address through conventional defense postures alone (15). NTS frameworks explicitly include threats to collective identity and culture as security concerns. The Copenhagen School’s concept of societal security defines this sector as threats to the sustainability of a society’s essential character, encompassing shared language, culture, religious practice, and national identity as objects of security in their own right (21). Neurotechnology, taken as a developing rather than fully realized capability, carries the potential to extend societal security threats into the cognitive domain.

As established prior, the broad definition of neurotechnology is operationalized in this article, which includes all tools, systems, and computational methods capable of recording, decoding, monitoring, influencing, or simulating neural activity, including invasive and non-invasive devices and algorithmic techniques that infer mental states, preferences, or cognitive patterns from physiological or behavioral data. These systems operate across three primary modalities, neuroimaging, neurostimulation, and brain-computer interfaces (BCIs), each carrying distinct NTS implications and each at a different stage of development, though all advancing toward operational capacity with sufficient momentum to treat their security implications as present rather than hypothetical. The account offered here is not a comprehensive technical inventory but is sufficient to establish the security logic the article advances. These modalities are inherently cross-operational, as most neurotechnology overlaps functional categories, and the dual-use potential is substantial, encompassing both the repurposing of civilian technologies for military or intelligence objectives and the reverse spillover of military-funded neuro-engineering into consumer markets (22).

At the individual level, the proliferation of these commercial neuro-platforms generates neural and neuro-adjacent data that could reveal mental states, preferences, emotional responses, and cognitive patterns without users’ awareness. This is, in the first instance, a privacy and autonomy concern. The NTS dimension emerges when that individual-level data is aggregated at the state population level. When the neural and behavioral profiles of large segments of a population are accessible to external actors, the conditions for influencing collective political cognition, shaping public deliberation, and manipulating the information environment in which political identity is formed become technically feasible. The threat is not to any individual mind in isolation but to the shared cognitive identity through which a citizenry forms political will, confers legitimacy on state institutions, and reproduces its capacity for collective self-governance. A state whose population is cognitively profiled, behaviorally predicted, and susceptible to externally mediated influence over political identity faces a security vulnerability that, while not without historical precedent in propaganda and psychological operations, is qualitatively distinct. It is not the manipulation of the information environment but the subtle shaping of decision-making processes at the level of individual neural architecture that reveals itself in the exercise of political agency.

Thus, the article’s primary construct is a system of systems - a continuous sequence moving from the individual cognitive system, through the collective cognitive system, to the state cognitive system, and from there to the international system of systems. Collective cognitive allegiance is a shared intersubjective structure that emerges from and is reproduced through individual cognitive identification, and it is this collective structure that constitutes the state as a political actor on the international scale (23). Neurotechnology, by reaching the substrate at the base of this chain, reaches the architecture on which political order at every level simultaneously rests. The Copenhagen School’s societal security framework provides the theoretical pathway from individual to state to international levels of analysis. The state’s authority and international standing rest, in part, on the coherence and autonomy of the political community it governs; societal security, as Buzan, Waever, and de Wilde (21) argue, is not reducible to state security but constitutes a precondition for it. If the cognitive substrate of that community is vulnerable to external actors, sovereignty is disrupted not through territorial violation but through the quiet destabilization of the political identity from which state legitimacy derives. This constitutes an NTS challenge that is at once domestic and international: domestic as it concerns the integrity of the governed population, and international because the actors holding disproportionate neurotechnological capabilities are predominantly foreign, their instruments are global, and the governance asymmetries they produce map onto existing hierarchies of technological power.

While a comprehensive account of emerging neurotechnological capabilities falls outside the scope of this article, the potential they introduce for recalibrating the identity foundations that underpin political authority at the state level is analytically consequential and cannot be dismissed as speculative. It is within this analytical frame that 2021 constitutional amendment acquires its security logic.

Cyber and digital sovereignty are constitutive elements of cognitive sovereignty, each operating at a different analytical level. They are fundamentally materialist frameworks, measuring capability, infrastructure control, regulatory reach, and economic capacity (24). What is at stake in them is the distribution of power over systems and data. Cognitive sovereignty, by contrast, analytically emphasizes not the distribution of material capabilities - though for the purposes of this article is analyzed through them - but the intersubjective processes through which identity, legitimacy, and norms are constituted and reproduced. Cognitive sovereignty responds to the disruption of the meaning-making processes within the state’s strategic population segments. Neurotechnology may well extend into competition for infrastructure control - as when the human mind is directly complemented by AI or computing systems through BCIs - but its defining threat is intervention in the constitutive processes of political identity itself, which is what sovereignty, at its cognitive foundation, depends upon. For the purposes of this article, however, the infrastructural and capacity materialist aspects that cyber and digital underpin are reduced to their structural core - the global power technological asymmetry and the infrastructural control and governance of neurotechnology that reaches into the cognitive domain.

### Sovereignty as rhetoric, and its limits in the Latin American context

From non-constructivist account, Weber (15), in analysis of the recalibration of sovereignty-related norms, shows that in addressing NTS, the principal concern has shifted from the protection of territorial sovereignty alone to the safeguarding of societies, communities, and individuals from threats that originate outside traditional state-on-state conflict. Examining this across the EU and the Asia-Pacific, she demonstrates that where human security remains unevenly institutionalized, sovereignty is routinely deployed as a semantic weapon, invoked strategically to resist perceived interference even when doing so obscures more complex interdependencies and forecloses effective policy responses. For Weber, this instrumental use of sovereignty is inconsistent with sovereignty’s absolute and categorical nature as she defines it, and ultimately counterproductive as the very power of the sovereignty claim becomes an obstacle to the NTS resolution it purports to protect. Weber’s conclusion is that progress on NTS requires altering actors’ perceptions of their available policy options and moving them beyond reflexive sovereignty rhetoric toward substantive engagement with the security challenge itself. Norms constrain state behavior but rarely determine it fully; security imperatives, economic interests, and domestic political considerations remain decisive. However, K. Weber’s cases are drawn from contexts of relative institutional capacity and technological parity, where sovereignty rhetoric functions primarily as a self-protective obstruction. This logic does not transfer straightforwardly to Latin America. In the Asia-Pacific, sovereignty is invoked defensively by states seeking to preserve existing strategies of maneuver and technological parity. In Chile and the broader Latin American region, where sovereignty discourse intersects with histories of US political and military intervention, post-colonial state formation, and sustained economic and technological dependency, the structural conditions are fundamentally different. Here, sovereignty is not a defensive posture adopted by relatively capable states; it is a claim asserted by a state whose practical autonomy has been repeatedly and materially undermined by external actors and foreign states. Weber acknowledges, following Acharya (25), that sovereignty norms are neither static nor historically predetermined, as norms of non-interference emerged during decolonization precisely to protect the authority and integrity of newly formed states against more powerful external actors. In this sense, sovereignty claims in the Latin American context may fulfill a different function than those Weber observes in the Asia-Pacific, not a counterproductive rhetorical reflex but a legitimate and structurally necessary assertion of agency against conditions of enduring asymmetry. Whether Chile’s 2021 constitutional amendment succeeds in navigating rather than reproducing the constraints of sovereignty rhetoric is what the following analysis seeks to examine.

### Sovereignty as performance: from territory to cognition

Weber’s (26) account is recasting sovereignty not as a condition states possess but as a performance they enact. Where Weber treats sovereignty as a legal absolute that states rhetorically invoke, Weber understands it as a performative construction, a condition sustained not by formal legal status but by repeated practices, narratives, and enactments that simulate the appearance of territorial and political integrity. Sovereignty has no fixed essential content; it acquires meaning through the norms that political societies attach to it and through the acts by which states demonstrate their authority over a bounded domain. In this account, sovereignty is always being made and remade. Where constructivism explains that sovereignty’s meaning shifts across time and context, C. Weber’s sovereignty-as-performance framework explains the mechanism. The meaning is produced and reproduced through iterative acts of political enactment, acts that must be repeated precisely because there is no stable foundation beneath them. This performative account is applicable to the sovereign claims states are beginning to advance in response to neurotechnological developments. As neurotechnology expands the reach of external actors into the cognitive domain of a population, through neural data extraction, behavioral profiling, and algorithmic influence, it reconfigures where state authority and integrity are meaningfully located within the state’s domestic affairs and international standing. The relevant site of sovereign assertion moves inward, from the territorial border to the cognitive boundary, the threshold between legitimate and illegitimate intervention into the mental domain of citizens. Just as intervention practices historically performatively reasserted, or as Weber terms it, ‘simulated,’ sovereignty in the geopolitical sphere, neurotechnology forces a rearticulation of sovereignty in the cognitive sphere. State sovereignty performance moves inward, giving rise to what this article proposes to call cognitive sovereignty, here understood as the state’s independent authority to regulate and maintain autonomous control over the mental and identity-related domains of its population, against foreign actors holding disproportionate capabilities for neural influence, data extraction, or cognitive manipulation. Sovereignty, in this sense, acquires meaning through the norms political societies attach to it; as neurotechnology reshapes what counts as permissible intrusion into the cognitive domain, it becomes a new site of sovereign performance, where autonomy is reasserted against neural and cognitive vulnerability associated with external influence.

### Cognitive sovereignty, technological colonialism, and self-determination

The risks that open the analytical space for the concept of cognitive sovereignty are examined in emerging scholarship through the concepts of technological and digital colonialism, used interchangeably here to denote forms of domination that survive the formal end of territorial colonialism and shift into non-territorial domains (27–29). These domains include the ownership of data infrastructure, the governance of algorithmic systems, and the extraction of human behavioral and neural data as economic and political resources. The concerns were articulated in Provision 24 of the first working draft of UNESCO’s Recommendation on the Ethics of Neurotechnology (56), which explicitly identified the need to address inequities in access to neurotechnology and to prevent ‘technological colonialism,’ understood as the use of technology as an instrument of colonization, producing bias, healthcare disparities, stigma, and cultural assimilation. Notably, the 2025 final version of the UNESCO Recommendation omits this language entirely (61). The omission may indicate institutional hesitation to provide this phenomenon with an explicit international governance framing, potentially leaving the normative work of contesting technological colonialism to be done at the national level. Yet the conceptual tools available at the national level are equally limited.

Traditional sovereignty concepts in international relations theory are organized around territorial and juridical categories, as formal legal recognition, exclusive jurisdiction over a bounded space, and the Westphalian norm of non-intervention. They can describe who controls land and who holds formal legal authority, but they were not designed to address interventions into the shaping of decision-making, the profiling of mental states, and the governance of deeper neural processes by external actors within another state’s population. It is this novel security risk that invites the concept of cognitive sovereignty, a framework capable of naming what is at stake when the domain being intervened with is not land or labor but the neural substrate of human agency and the collective cognitive identity of a state’s population. In this sense, cognitive sovereignty may derive its normative foundation from the right to self-determination - a right that emerged in the context of territorial decolonization but has been acquiring new meaning as forms of interference shift into the cognitive domain.

The Declaration on the Granting of Independence to Colonial Countries and Peoples (1960) (57) framed self-determination as a universal right, but its adoption was decisively shaped by the political imperatives of territorial independence and decolonization. International legal doctrine subsequently confined the right’s application primarily to colonial peoples, assuming that those within already-constituted states had, by virtue of statehood itself, already exercised it. Subsequent legal scholarship has sought to reframe self-determination by distinguishing between ‘external’ self-determination, the right to full political independence from a larger state, and ‘internal’ self-determination, the continuous right of peoples to govern themselves freely within already-constituted states (30).

If internal self-determination is understood as an ongoing entitlement of a people to constitute their collective identity and governance free from external domination, then the emergence of technological instruments of external influence, neural data extraction, algorithmic behavioral governance, and AI-mediated profiling, constitutes a direct challenge to these collective rights. The right to self-determination may therefore be amenable to renewed reinterpretation in the contemporary neuro-digital landscape, extending its protective scope to cover forms of cognitive dependence inconceivable at the time of decolonization, claimed via collective rights of peoples to form independent governance over their politically relevant cognitive identity.

Within this reinterpretation, cognitive sovereignty can be understood as emerging along two analytically distinct but related pathways. First, it arises as a reactive sovereignty claim, articulated in response to perceived external cognitive interference, where the state asserts authority to protect the population’s cognitive domain from intrusion, manipulation, or extraction. Second, it emerges as a constitutive expression of internal self-determination, where a political community claims the right to shape, preserve, and govern its own collective cognitive identity from within, independent of external structuring forces. In this sense, cognitive sovereignty is not only a defensive response to interference but also an affirmative claim grounded in the continuous right of peoples to constitute themselves as a political community. It represents the institutionalization of this claim through domestic state authority in the neuro-digital sphere, translating the normative principle of self-determination into governance over the cognitive conditions that sustain political identity and legitimacy.

## Analytical framework and hypotheses

This article’s central analytical proposition is that Chile’s 2021 constitutional neurorights amendment can be functionally interpreted as acquiring the structure of a cognitive sovereignty assertion in response to NTS challenges arising from global neurotechnological and digital asymmetries. This reading does not claim to demonstrate the original intentions of the amendment’s drafters but advances an account of what the amendment may do, whether or not those functions were consciously intended. Three contextual dimensions support this reading; their combination challenges the view that the amendment (H0) is adequately explained as a rushed, conceptually underdeveloped liberal rights extension.

H1 proposes that the amendment’s negative-rights framing, its placement within the right-to-life provision rather than the cognitive-autonomy clause, and its coexistence with Chile’s restrictive reproductive-rights framework collectively suggest that the individual-autonomy reading is incoherent and that the amendment is inconsistent with a liberalizing rights logic. H2 proposes that Chile’s history of foreign political intervention - US-sponsored destabilization and the Pinochet regime’s systematic use of cognitive methods to fracture political consciousness – constitutes a historical context within which the amendment’s non-interference logic acquires cognitive sovereignty resonance, and foreign non-interference security incentive. H3 proposes that Chile’s structural position as a consumer rather than producer of neurotechnology, operating under conditions of longstanding economic and technological dependency, that the literature identifies as technological colonialism, provides the geopolitical context within which the amendment may function as a reverse regulatory claim, asserting Chilean authority over foreign neurotechnological actors operating within its territory. Considered together, these three contextual dimensions support a reading of the amendment as a strategically meaningful sovereignty instrument, challenging the adequacy of H0 as a complete account – not by demonstrating that the amendment was designed as a sovereignty assertion, but by showing that H0 fails to account for the provision’s structural characteristics, and geopolitical function.

## Neurorights in existing human rights frameworks: refuting H0

### Neurorights within the human rights framework

The dominant position in the existing neurorights literature is that established human rights, to bodily integrity, privacy, personal identity, freedom of thought, and autonomy, already apply to most foreseeable neurotechnological scenarios, rendering a dedicated category of neurorights largely redundant on the individual level of protection (31). Some scholars have nonetheless argued that these protections may prove insufficient for the emerging neurotechnological landscape and have proposed an additional layer of safeguards specifically addressing cognitive liberty, mental privacy, psychological continuity, and mental integrity against unauthorized access, manipulation, or data extraction (32). This debate gave rise to neurorights as an umbrella term for a proposed set of rights intended to ensure adequate protection of the human mind and brain.

It is necessary to distinguish between neurorights as moral claims and neurorights as legal instruments. Legal rights are not merely legalized versions of moral interests; they are technical constructs embedded within specific legal frameworks, interrelated with other rights, institutional mandates, and constitutional norms. Within constitutional and law-enforcement systems, rights function as part of a broader conceptual architecture, and in the context of neurotechnology, that architecture determines how neurotechnological capabilities are situated within or constrained by the framework of state independent governance and sovereignty. When the lawfulness of state action requires balancing human rights against state interests, state domestic authority as sovereignty is operationalized through national-security or public-interest justifications. Human rights are weighed in absolute or qualified terms, with deference to state interests calibrated accordingly. Understanding neurorights therefore requires situating them within this legal balancing framework rather than treating them solely as moral and legal individual entitlements. As Ligthart et al. (33) emphasize, the mere declaration of neurorights, whether at the constitutional level or in international soft law, does not by itself render such rights operational or enforceable. Neurorights must ultimately be ‘translated into actionable regulatory mechanisms’ and integrated within a system of duties, enforcement procedures, and interpretive constraints; otherwise, they risk remaining symbolic or conceptually indeterminate. Istace (34) similarly demonstrates that the existing human-rights architecture, encompassing rights to privacy, bodily and mental integrity, and freedom of thought, already provides a flexible framework capable of extension through evolutive interpretation, but only where domestic courts and legislatures actively apply such protections in concrete cases.

As Ienca and Andorno (32) and Istace (34) show, neurorights as currently theorized would operate across two tiers within the international human-rights framework. At the absolute tier, encompassing human dignity, personhood, and the freedom of thought, neurorights reinforce the inviolability of thought and mental integrity against any interference, whether by the state or private actors. At the qualified tier, encompassing rights to privacy and freedom of expression, which may be lawfully limited by state interest, neurorights strengthen mental privacy as a fundamental but limitable safeguard. As part of the equality-of-rights principle, they would further imply a positive state duty to ensure equitable, safe, and inclusive access to neurotechnology. In this way, neurorights seek to connect established human rights to emerging technological realities, reshaping legal conceptions of autonomy and dignity to encompass the cognitive and neural dimensions of personhood within the state domestic governance architecture. Yet their precise location within this framework remains contested and, as stand-alone constructs detached from enforcement mechanisms and interpretive practice, their legal determinacy is limited.

Understanding neurorights therefore requires moving beyond the question of whether they are morally justified to ask how they function within a legal balancing framework, and crucially, whose interests they serve when that balancing occurs, particularly when evaluating domestic and international level interests. Where neurorights remain unenforced at the domestic level, they cannot be assumed to carry the same automatic operational weight at the international level either. Yet when neurorights protections are overridden by national security interests within a state’s domestic governance architecture, their assertion at the international level functions not as a legal claim but as a political strategy, a deliberate act of sovereign positioning in the neuro-digital sphere rather than a domestically enforceable entitlement.

### The constitutionalization of neurorights in Chile

Chile’s 2021 neurorights constitutional amendment emerged through the Senate’s Comision de Desafios del Futuro, Ciencia, Tecnologia e Innovacion, an institutional body created to anticipate and legislate on frontier scientific, technological, and societal transformations. The Commission’s mandate encompasses long-term technology governance and scientific autonomy for Chile’s socioeconomic development, oriented toward anticipatory governance of emerging technologies rather than reactive regulation. This institutional pathway positioned neurotechnology governance as a matter of national strategic interest.

The amendment entered the constitutional debate when Senator Guido Girardi engaged Rafael Yuste, the proponent of the US BRAIN Initiative and founder of the NeuroRights Foundation, to testify before the Commission and advise on legislative drafting. Girardi’s push for strict non-interference neurorights, despite expert public concerns (35), received unanimous parliamentary support in December 2020. The constitutional amendment was signed into law, however a statutory bill seeking to operationalize the amendment through defining neurodata, setting limits on neurotechnological interventions, and establishing safeguards for research and commercial applications was proposed in parallel but remains stalled. This legislative inertia shows that the amendment functions as normative guidance rather than an operational regulatory regime. The provision is formulated primarily in negative, non-interference terms and prohibits specified intrusions into neural functioning and neural data but does not create positive state duties, regulatory infrastructure, or guaranteed access to neuroprotective safeguards.

The most significant domestic legal development since the amendment’s adoption is the 2023 Supreme Court’s decision in *Girardi v. Emotiv* (Rol No. 105.065–2023) (55), in which the constitutional provision was applied to a dispute involving a consumer neurotechnology company. The Court’s reasoning proceeded entirely within the framework of individual rights, personal mental privacy, and data protection – not through state-authority framework (36). This development, on the one hand, challenges the sovereignty-asserting function this article advances as not being the provision’s operative legal function, and reinforcing human-rights framing. On the other hand, the decision may be read as consistent with a limited protective-sovereignty account. The Court, by applying this constitutional provision, de facto extended Chilean jurisdictional authority into the cognitive domain. The reading requires that the functional effect of the provision, as applied, is to assert Chilean state authority over the conditions under which foreign neurotechnological actors may reach the cognitive domain of Chilean citizens.

However, what is not enforceable at the domestic level may still function as a political strategy at the international level, reflecting deeper political orientations and historically rooted concerns. Collective political memory, shaped by experiences of foreign intervention and institutional legacies, can guide decision-making in ways that are not immediately legible, but become more intelligible when situated within a longer political and historical trajectory.

What matters here, therefore, is not domestic enforceability but international legibility and the act of constitutional assertion can be read as a sovereign signal. What may appear underdeveloped from a domestic human-rights perspective may nonetheless be strategically coherent as an international sovereignty claim. In such cases, the state asserts constitutional authority over the cognitive domain without fully operationalizing it, where the act of assertion itself constitutes the primary political objective. The next section demonstrates that neurorights, in the Chilean case, are disproportionate to the existing governance ecosystem, thus they should not be understood as a purely domestic project.

## Incompatibility of neurorights as extension of autonomy and the normative bioethical ecosystem in Chile (H1)

In Latin American context, bioethics is understood in close relationship with human-rights protection, the promotion of democracy, and civic engagement (37). Chile’s bioethical discourse reflects these regional characteristics while carrying historically specific tensions shaped by the moral authority of the Catholic Church, the legacy of authoritarian governance, and post-colonial patterns of institutional conservatism. That normative environment is structurally resistant to the expansion of individual autonomy, which makes the emergence of neurorights within it not straightforwardly explicable as a liberalizing move.

### Structural contradiction of neurorights

Evidence that the 2021 constitutional amendment may be functionally operative beyond a focus on individual autonomy lies in the tension between what neurorights ostensibly protect and what Chile’s broader governance constrains. Chile currently permits abortion only on three grounds: risk to the life of the pregnant person, fetal non-viability, and rape or incest, placing it among the region’s most restrictive reproductive-rights regimes (38). Decisional autonomy, bodily integrity, and the permissible extent of state intervention in decisions of profound personal consequence are precisely the domains 2021 constitutional amendment claims to safeguard in the cognitive register. Neural decision-making is elevated to constitutional status while bodily decision-making remains constrained, a contradiction difficult to explain if the 2021 constitutional amendment is read as a genuine commitment to individual autonomy.

The constitutional architecture deepens this tension. The neurorights amendment was incorporated into Article 19, Number 1, the very provision guaranteeing the right to life and physical and psychic integrity that simultaneously contains the protection of life before birth. The structural proximity of neurorights to the right-to-life clause raises the question of whether this placement was intentional, signaling that neurorights operate only in relation to interferences that threaten ‘life’ as defined by Chile’s existing constitutional doctrine, rather than marking an independent expansion of cognitive autonomy as a distinct constitutional value. Porter (39), from a theological-legal perspective, argues that the extension of the right to life to include a cognitive layer ultimately roots governance in an understanding of the state as the conduit of authority over life in all its dimensions, including the brain and its data, which supports the view that neurorights, even when framed as protections of the population, function as an extension of state authority. The 2022 constitutional draft, which would have significantly expanded neurorights by establishing active protections rather than merely prohibiting interference, was rejected in the national plebiscite, partly due to conservative Catholic mobilization against its provisions expanding reproductive autonomy and redefining family structures (40). The 2021 amendment, by contrast, did not challenge these inherited structures and permitted the state to assert constitutional governance over the brain without disturbing the conservative normative order that surrounds it. As Kottow and Russo (37) observe, ‘conservative forces slow down any attempts to revise moral values, even when the status quo is damaging and unwanted.’ The different outcomes of the two constitutional interventions suggest an underlying political logic, where governance over the cognitive domain was more acceptable because it did not expand individual autonomy in ways that might challenge the Church’s institutional authority or the state’s existing governance of moral life.

### The negative-rights framing and its internal contradictions

Further evidence that the 2021 constitutional amendment may be incomplete when viewed solely as a rights-expanding project lies in the structural limitations of its negative-rights framing. As Fins (41) argues, the dilemma is sharpest in cases of minimally conscious state and cognitive-motor dissociation, conditions in which neurotechnology can detect or restore covert cognition and communicative capacity in ways traditional bedside assessment cannot. Chile’s negative-rights framing risks foreclosing these interventions as a purely non-interference framework may prevent the identification of covert consciousness, impede diagnostic innovation, and obstruct neuroprosthetic technologies capable of giving communicative voice to those who are neurologically silenced, potentially conflicting with the UN Convention on the Rights of Persons with Disabilities (58). Negative-rights neurorights protect individuals from interference but do not guarantee the capabilities necessary to exercise autonomy, a structural incompleteness that extends to Chile’s broader governance pattern in which bodily and cognitive agency are consistently overridden at the moments when autonomy is most consequential. Neurorights, framed as non-interference, do not disturb these patterns; they reproduce them in the cognitive register.

Understanding the neurorights agenda therefore requires, after showing its internal incompatibility with the domestic bioethical account, situating it within broader post-colonial patterns and the history of foreign intervention that enabled the rise of a regime responsible for gross human-rights violations, including forms of targeting the cognitive domain outside of explicitly neural terms. These practices raise a question that carries into the neurotechnological present, namely whether the state of Chile holds the authority to define, protect, and govern the cognitive domain of its people, rather than external actors.

In Chile, this question is inseparable from a historical continuum of contested sovereignty, first as a post-colonial territory and later as a state heavily affected by foreign intervention, and it is this continuum to which the following section turns, focusing on the Monroe Doctrine.

## The fractured soul: foreign intervention and cognitive sovereignty (H2)

“*There are three sources of power in Chile,” one Chilean intelligence officer informed a U. S. military attaché in early 1974: “Pinochet, God and DINA*. (52, 59)”

Chile’s political consciousness of sovereignty was formed under conditions of systematic foreign interference. The Monroe Doctrine, originally announced in 1823 as a diplomatic warning against European intervention, was progressively reinterpreted as a mechanism for US hemispheric dominance. Successive reinterpretations from the Roosevelt Corollary to Cold War containment enabled political, military, and economic intervention across Latin America, inverting the Doctrine’s original meaning, where non-interference became unilateral enforcement authority, embedding a structural hierarchy between the US and Latin America (42). Early national documents recognized that Latin American states ‘cannot in isolation defend their sovereignty,’ requiring collective hemispheric resistance to European hegemony (43). The Monroe Doctrine evolved as a response to oust European colonial power in the region but quickly transitioned into a patronistic power umbrella and an assertion of US implicit governing authority. In Chile’s case, this trajectory culminated in the 1973 overthrow of the democratically elected Allende government. Nixon and Kissinger authorized CIA destabilization operations, including propaganda, economic pressure, and support for opposition forces, out of concern that Chile would become ‘another Cuba’ (43). Declassified documents reveal ongoing CIA support for the Pinochet regime, including engagement with DINA, despite knowledge of systematic human-rights abuses.

The Pinochet regime, facilitated by foreign intelligence intervention, brought political repression that was specific in the cognitive nature of its methods. As documented in the Rettig Report and the Valech Commission findings, DINA’s practices were designed to fracture consciousness through sensory deprivation, forced isolation, mock executions, manipulation of temporal perception, and psychological techniques intended to disrupt the mental architectures through which individuals formulate beliefs, participate in political life, and exercise autonomous agency (44). Survivors reported lasting cognitive impairments, trauma-induced memory fragmentation, dissociation, and long-term alterations in decision-making and emotional regulation. This was a political repression which entailed systematic dismantling of cognitive autonomy at both individual and collective scales, producing what filmmaker Patricio Guzmán termed Chile’s ‘fractured soul’.

The sovereignty question was then renegotiated by Chile’s population in response to Pinochet’s arrest in London in 1998, which functioned as a transformative “memory knot.” By permitting criminal proceedings abroad and later pursuing domestic legal cases, Chile accepted a disruption of traditional head-of-state sovereign immunity in favor of accountability, recovering damaged sovereignty by momentarily ceding it to universal jurisdiction. Chile’s sovereignty is thus not simply territorial or institutional; it is tied to the restoration of collective moral and legal order and collective agency over autonomous political decision-making. The 2021 constitutional amendment should be read within this context as a constitutional claim to govern the cognitive domain that was once violently expropriated by a foreign-installed regime. What began as a political sovereignty claim has evolved into a cognitive sovereignty claim, the capacity of a society to act freely upon its past, and to insulate the mind against new NTS threats posed through emerging neurotechnological hegemonies and interventions.

## Asymmetry as a security claim in search of symmetry (H3)

### Chile’s structural position in the global neurotechnology economy

Deeper structural economic dependencies predated and were intensified under the Pinochet regime. Decree Law 600 (DL 600; 1974 to 2016), promulgated under the dictatorship, created virtually unrestricted foreign direct investment, institutionalizing a model in which foreign capital penetrated Chile’s economy without reciprocal state capacity to regulate technological or infrastructural dependencies (45). This model outlasted the dictatorship itself. Chile achieved substantial post-Pinochet economic development, becoming the first Latin American country to join the OECD in 2010. Yet in digital and neurotechnology domains, Chile remains primarily a consumer: importing equipment, hosting international data centers, and servicing global platforms rather than generating them domestically. In 2023, only 11.7% of Chilean adults achieved technological proficiency, against an OECD average of 32.3% (46); R&D investment stands at approximately 0.4% of GDP, against an OECD average of 2.7% (47).

The global neurotechnology landscape is dominated by the North America (roughly 54.9% of the global neurotechnology device market in 2024), followed by Europe and Asia-Pacific actors. Chile’s domestic neurotechnology market is estimated at approximately USD 183 million, modest and dependent (48). Chile has become the world’s neurorights pioneer despite lacking the domestic technological infrastructure that such rights claim to govern, which could demonstrate that the state is attempting to regulate a domain in which technological power lies overwhelmingly outside its borders.

### Digital colonialism and the reverse Brussels effect

Revisiting Weber’s (15) sovereignty framework, sovereignty recalibration in Chile does not emerge from technological competition but from technological dependence. Even when sovereignty claims are dismissed as semantic, this does not negate that technological dependencies materially constrain the capacity to exercise sovereignty in practice. Under these conditions, sovereignty claims acquire heightened strategic importance as attempts to recalibrate state political agency in the face of structural constraint. Mery (49) identifies digital colonialism as the mechanism through which this plays out for Chile. Digital colonialism describes the global economic order grounded in data extraction and platform ownership by dominant powers, particularly US Big Tech, whose software ecosystems and cloud infrastructures keep countries like Chile structurally dependent. Chile’s own data protection legislation was also shaped under direct consultation with EU officials, exemplifying how regulatory models circulate asymmetrically through what the literature calls the ‘Brussels Effect.’ Both technological and regulatory forms of digital colonialism operate simultaneously (49). Chile’s 2021 constitutional amendment can thus be read as an attempt to generate a reverse Brussels Effect, as a pushback to digital colonialism, in the neurotechnological domain, in which it establishes regulatory precedent that dominant actors must engage rather than impose, an inversion of the usual asymmetry.

### Where territorial and cognitive sovereignty converge

In parallel to the reform, Chile has become an attention point for the territorial, environmental, and political battles provoked by AI infrastructure. Data centers have triggered protests over water consumption, land use, and the displacement of local communities (50). Google’s proposed data center in Cerrillos, requiring an extraordinary volume of water per second for cooling, shows how digital infrastructures reproduce extractive logics long characteristic of colonial and neocolonial economies (50). Conflicts surrounding Amazon and Google data centers, perceived as environmentally destructive and socially extractive, have already led to local protests and cancelations of planned facilities (49). Chilean officials warn that AI risks being treated as a new ‘fetishism,’ with data centers given priority over citizens (50). Chile’s sovereignty claims in technology additionally became on the same presidential agenda with its renewed territorial claims. In 2025, Chilean President Gabriel Boric became the first sitting head of state to visit the US-run Amundsen-Scott South Pole Station, framing the visit as a reaffirmation of Chile’s sovereignty claims in Antarctica (51). Chile is thus renegotiating sovereignty across physical, digital, and cognitive terrains simultaneously, and the 2021 constitutional amendment functions as a normative-political instrument for that renegotiation. This dynamic may prove not to be unique to Chile, but rather to reflect a structural condition shared across the Global South, where states simultaneously host the infrastructure of technological power while remaining excluded from its governance.

## From 2019 protest symbolism to the cognitive sovereignty claim: synthesis

The analytical threads additionally revealed in the collective right of self-determination and cognitive sovereignty converge in the symbolism of the 2019 estallido social. The ratification of the neurorights amendment coincided with a political atmosphere shaped by mass mobilization, collective frustration with dictatorship-era economic structures, and demands for constitutional renewal. The protests were deliberately timed to coincide with October 18, ‘18–10,’ a date that, read as ‘1810,’ marks Chile’s independence from Spanish colonial rule (39). Protest chants and artworks demanded autonomy over mind and body as extensions of Chile’s long-standing quest for self-determination, framing the neurorights movement within Chile’s broader historical narrative of liberation from external domination.

Chile inherited a Catholic, colonial, and post-authoritarian understanding of sovereignty rather than a reconfigured secular one. The state continues to perform sovereignty as both political and moral guardianship, a tradition that constructs the sovereign as a secular analog to divine authority, as the indivisible source of authority who retains the power to decide the exception. If state sovereignty is understood as an earthly continuation of divine authority, then constitutionalizing the right to life into the cognitive register through neurorights extends this theological model. In this reading, neurorights become an instrument of sovereign power modeled on the unity and indivisibility of divine authority, transposed into the realm of cognitive guidance and exercised through what Hobbes called the ‘Mortal God’ of the sovereign state.

By articulating this continuity, the Chilean case illuminates how cognitive sovereignty becomes a moral and constitutional imperative, a lived political expression of independence aligning the language of rights with a historical quest for statehood independence from external interference. Populations through state’s historical conscience, shaped by successive experiences of colonial extraction, foreign-sponsored regime change, systematic cognitive disruption, and digital dependency, can evolve into a constitutional conscience, transforming the collective memory of subjugation into a shared constitutional principle of protection against future technological encroachment.

## Discussion

The three contextual dimensions developed above converge to challenge the adequacy of H0 and support the cognitive sovereignty functional reading. H1 suggests that a liberalizing autonomy reading of the amendment faces an internal tension: it emerged within, and did not disturb, a normative order in which the state asserts governance over moral and bodily life through conservative Catholic-inflected frameworks, and it is ineffective as a domestic policy project. The placement of the amendment within the right-to-life provision, rather than in a distinct cognitive-autonomy clause, signals the extension of existing sovereign governance rather than the inauguration of a new rights paradigm. H2 supports this interpretation by establishing the historical conditions under which a cognitive sovereignty reading becomes contextually intelligible, and shows the state non-interference security standpoint; the cognitive dimension of sovereignty is not a novel concern for Chile but a site of lived violation, exercised through Monroe Doctrine authorized foreign intervention and DINA’s methods, which were designed to fracture the mental architectures through which autonomous political agency is exercised. The amendment may be read as a constitutional response to the possibility of that fracture recurring through technologically mediated external means, even if not intended as such. H3 situates this reading within the structural asymmetry that gives it geopolitical weight and that makes it strategically relevant, as Chile is attempting to regulate a technological domain in which economic power lies overwhelmingly outside its borders, in circumstances that reproduce the extractive dynamics of its colonial and neocolonial past. Considered together, these three contextual dimensions propositions challenge the adequacy of H0 as a complete account.

When asserted against the *Girardi v. Emotiv* decision, it is evident that the Court did not invoke the provision’s operative legal function as a sovereign assertion, yet the decision de facto instantiated that function at the level of the regulatory effect, independently of any explicit doctrinal intention.

The broader strategic utility of this framing extends beyond Chile. The analytical model forecasts the uptake of cognitive sovereignty reassertion via potentially constitutional neurorights among states with two structural characteristics: limited domestic neurotechnology capacity, and a relationship of technological asymmetry or strategic competition with dominant actors such as the US or EU. Under these conditions, constitutional neurorights function not primarily as individual protections but as pre-emptive sovereignty instruments, anticipatory governance measures aimed at counterbalancing technologically dominant powers. This logic may forecast cognitive sovereignty instruments adoption via neurorights for technologically asymmetric states of the Global South, and potentially via a different normative framework within states in technological competition, such as Russia and China. Several states in Latin America are already pursuing similar initiatives, though not yet at the constitutional level. The emergence of neurorights in international governance debates should therefore be read not merely as ethical progress but as a new area of global power contestation and security in which the cognitive domain of populations has become a stake of sovereignty.

## Limitations and future directions

This article acknowledges several significant limitations. The constructivist and critical theory examination of shifting state sovereignty warrants deeper review in light of the regulatory predictions advanced here and the treatment of neurorights as a security instrument.

Most critically, the sovereignty argument as presented is structurally plausible rather than empirically demonstrated. Establishing it rigorously would require process-tracing of how political memory actually translated into legislative action, including whether the legislative deliberations show evidence of sovereignty-oriented framing choosing the rights-oriented pathway. Deeper engagement with Chile’s privacy jurisprudence across periods of political instability, and examination of the statutory reforms now stalled post-amendment, would substantially strengthen or complicate the argument. A technically informed assessment of the actual neurotechnological risk landscape in Chile is also necessary to show that the security and sovereignty claim is most compelling if the neurotechnological threats it purports to address are real and proximate rather than speculative. Engagement with the Inter-American human-rights system is further necessary to understand how regional legal norms may shape or constrain neurorights-related developments. Finally, a broader comparative study of other Global South states pursuing neurorights-adjacent legislation would test the generalizability of the sovereignty-reframing model and move it from preliminary framework toward a robust analytical theory. These constitute the primary directions for future research arising from this article.

A further methodological limitation concerns the movement between levels of analysis. The article operates simultaneously at three levels: the individual, where neurorights function as protections for personal cognitive liberty; the state, where the article reframes them as instruments of cognitive sovereignty over a population’s mental domain; and the international, where state constitutional assertions are read as strategic positioning within global technological power structures. These levels are not automatically connected, and the theoretical mechanisms linking them are assumed rather than strongly theoretically demonstrated. What aggregates individual cognitive vulnerability into a state-level security concern is not self-evident; the claim that discrete neural risks to individuals translate into collective political vulnerability at the population level requires an account of how cognitive interference scales. Similarly, what makes a constitutional assertion by a structurally dependent state internationally legible as a strategic signal, rather than purely domestic symbolism, requires more careful specification than this article provides. Future research should explicitly theorize these cross-level mechanisms, engaging the aggregation problem between individual and collective cognitive harm, and the conditions under which state-level normative assertions acquire strategic weight in the international system. Addressing these gaps is necessary to move the cognitive sovereignty framework from conceptual proposal toward a theoretically grounded and empirically testable proposition. A related and pressing area for future research concerns international cognitive meddling as an emergent security practice. The capacity for external actors to influence the political cognition of foreign populations is not new; information operations, propaganda, and psychological influence campaigns have long histories. What neurotechnology introduces is a qualitative shift in the precision, scalability, and depth of that influence, moving from the manipulation of information environments to the potential profiling and targeting of individual neural and emotional states. This dimension of cognitive interference, already visible in the intersection of AI-driven behavioral prediction and consumer neurotechnology, has not yet been systematically theorized as a category of international security threat. Its implications for sovereignty, international security and state independent governance, require dedicated scholarly attention, and this article’s framework offers one analytical starting point for that inquiry.

## Conclusion

The individual’s mind, long treated as the inviolable site of human agency, emerges in this analysis as a new arena in which state sovereignty and global power competition are both challenged and reasserted. 2021 constitutional amendment when examined against the country’s historical experience of violated sovereignty and modern security concerns, its bioethical architecture, and its structural position within global technological asymmetries, reveals a sovereignty logic that the human-rights framing obscures. As neurotechnology proliferates and reconfigures power asymmetries, cognitive sovereignty is likely to become an increasingly salient security objective, which would be asserted through variety of normative tools, which require investigation. The redundancy arguments leveled against neurorights within human-rights frameworks, rather than settling the question, reveal that such a governance move cannot be dismissed simply because it does not achieve the individual-protection objective it nominally declares. Whether or not Chile’s architects intended it as such, the 2021 constitutional amendment exhibits the structural characteristics of a state sovereignty assertion. The ex post functional reading this article advances depends on demonstrating the amendment’s coherence within its historical, geopolitical, and NTS context. This reading carries strategic implications of neurotechnology governance in the Global South and beyond.

## Data Availability

The original contributions presented in the study are included in the article/supplementary material, further inquiries can be directed to the corresponding author/s.
